# Supplementary figures and images for: Two novel mutations in MSX1 causing oligodontia
Source: PLoS One. 2020 Jan 8;15(1):e0227287. doi: 10.1371/journal.pone.0227287 (PMC6948825; doi:10.1371/journal.pone.0227287)

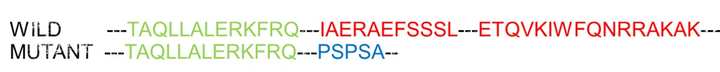

Supplement: S1 Fig — (TIF) [file pone.0227287.s001.tif]

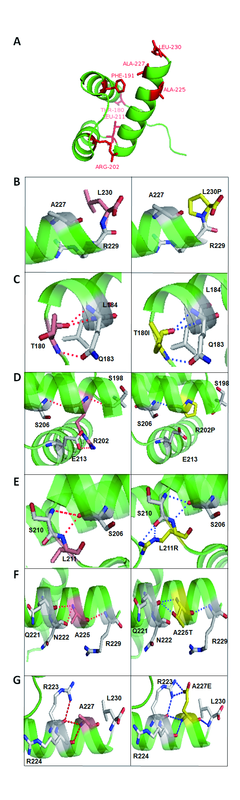

Supplement: S2 Fig — Thr180Ile and Leu230Pro all caused less than 6 missing teeth, neither mutation seems to cause any obvious changes in the structural interface, possibly explaining the milder effects. The other mutations all caused more than 6 missing teeth and had alterations in hydrogen bond formation. The alterations may lead to changes of the helical conformation, ultimately resulting in an alteration of protein folding and decreased stability. (A) Locations of the mutational sites on the structural model of human MSX1 homeodomain structure (MSX1 homeodomain green, mutational sites red). (B)- (G) Pair-wise comparisons between the wild-type (left) and mutant (right) residues for predicted changes in local contacts with other amino acids. The salmon stick models indicate wild-type residues, yellow indicate mutated residues, gray indicate neighboring residues, with the short bar in white, red and blue standing for the carbon, oxygen and nitrogen atoms, while the dashed lines in red and blue illustrated the hydrogen bonds. Here, we define a hydrogen bond geometrically as having a donor–acceptor distance ≤3.3 Å. The hydrogen bond distances are determined by Swiss-pdb Viewer. (B) Leu230 and the Leu230Pro mutation both do not have hydrogen bond interactions with adjacent residues. So, the mutation does not seem to cause any obvious changes in the structural interface, possibly explaining the milder effects found for this mutation. (C) Thr180 is predicted to have hydrogen-bonding interactions with Gln183 and Leu184. The Thr180Ile mutation does not alter hydrogen-bonding with these two amino acids. (D) Arg202, located in the α helix II, forms hydrogen bonds with adjacent residues Ser206 located in the same α helix II and Ser198 in the loop and Glu213 in the α helix III. The p. Arg202Pro mutation is predicted to abolish the hydrogen bonding with Ser198 and Glu213. (E) Leu211 is predicted to have hydrogen-bonding interaction with Ser206 located in the same α helix II. The mutant protein has [file pone.0227287.s002.tif]
