# Supplementary material for: Two novel mutations in MSX1 causing oligodontia
Source: PLoS One. 2020 Jan 8;15(1):e0227287. doi: 10.1371/journal.pone.0227287 (PMC6948825; doi:10.1371/journal.pone.0227287)
Supplement: S1 Table — (DOCX) [file pone.0227287.s003.docx]

**S1 Table Primers for candidate genes**

| ***MSX1*** | 5’ → 3’ | |
| --- | --- | --- |
|  | F | R |
| 1 | CTTCAGCGCAGAGGAAAGT | AAGAAGTCATGTCAGCAGCC |
| 2 | GCCTCGCCTTATTAGCAAGT | GAGCGAAGGGGACACTTT |
| 3 | GGCTGCTGACATGACTCTT | TGGGTTCTGGCTACTCACTG |
| 4 | GCGGCACTCAATATCTGGTA | ACATGGGCCGTGTAGAGTC |
| 5 | AGCTGGAGAAGCTGAAGATG | CATGGCCTCTAGCTCTGTTC |

| ***AXIN2*** | 5’ → 3’ | |
| --- | --- | --- |
|  | F | R |
| 1 | AGAAATCAGAACTCGGGCTGAGA | CGAGCTTCCACCCCCACCTT |
| 2 | TCTCTCTCCCCACCTCCCCC | TCCACGCATTTCTCCCTCTCCA |
| 3 | CGGATTCCCCTCTGACCCGG | GTCCACAGCATCAGCCCACC |
| 4 | CAGCAGGTTGGCGTTGAGCA | TCCACTCCCAAGCAAGCCCA |
| 5 | TCCCTGTGGGAGCACCGATG | TCCCTTTGCCCTAGGGGACC |
| 6 | CCCGAACATGGGTTTGCGGT | ACACATACGAGCGCTCACGC |
| 7 | CTCCTGCGTAGGGAGCCGAA | ACCTGGCTGGGAGACAAGCC |
| 8 | GGGAGCAGGTGCATGGGTCT | TGTGGTCTGCTCAGTCCAACGT |
| 9 | TCCAGTTCTTCTAACCCAGTTTCT | CCAGGTCTCCACCCAAACCCA |
| 10 | ACCCAGGGTCTTGGTTGGGT | ACTGTTCCTCATCACTAGCGCT |
| 11 | GCGCACGTGTGTGTTTGCTT | CACCCTGCCCCACCTAGGTC |
| 12 | TTGTGGTGTGGCCAGTCCCA | TGGATTTCAGATCCCTAGGAAGTCA |
| 13 | GCTGCCTGTATTTGAGAGACTGCC | GGCCTGGGGAGGCTACATGA |
| 14 | GGGCCCCAGGTTGATCCTGT | CGGGTGACCGTGCAGGTACA |
| 15 | TCCACTATCTCTAGTGCTTGACTTT | CCTGGCCGGACTGGAGACAA |

| ***EDA*** | 5’ → 3’ | |
| --- | --- | --- |
|  | F | R |
| 1 | AGGCAGAGGCGAACCCTCAC | GCAACTCCGAGCGCAACTCT |
| 2 | CGAAGGGAACAGCTGCCTGC | GGCTCGAGGTTGCTGAGTGC |
| 3 | TGGCTATGACTGAGTGGGGTCA | ACCTTGGGTCTGTGGTGGAC |
| 4 | TCTTGGGGATCCCTCCTAGT | TACAAAAATCGCACTCTTGATTT |
| 5 | TCCTGACAGTACACTCATCACAGGA | GCACCTGGTGGGGTGGAGAG |
| 6 | GTGCACTCTGACTCTTCCTCCA | CTACCCAGGAAGAGAGCAATCC |
| 7 | CCATTACTCATAGTGACTACTCT | GATTATTTGGAGGCTGGGGAG |
| 8 | TGTTGCCTCGATTATTCTGACA | CACCGGATTTGCATTTTGGATA |
| 9 | TTCTGTCAATTCACCACAGGG | TTAGAGGTTCTGGGAGTCCTGGC |

| ***PAX9*** | 5’ → 3’ | |
| --- | --- | --- |
|  | F | R |
| 1 | CGCTAATATGGGGAAACTGA | ATTGGGGATGGAGACAGC |
| 2 | TCCCTTCGAGTCATTCACAT | CAGACAGAAACTTTCCGCAC |
| 3 | TTTGCCCTCTCGCCTCCTCC | CATTGAACTGCTGTGCGCCG |
| 4 | GGTTTGGGGACAGCCCCAGT | TGTACTTGTCGCACACGCCG |
| 5 | CACCGTGGTGAAACACATC | GGAAAGACAGTGTCCCTGAG |
| 6 | GTCTAAGCCCTCCAGCTCTC | ACGAAGGATCTGGCTCGTA |
| 7 | CAGAGCATTGCTGGCTTACT | AGGATGTGAGTCCGTACAGC |
| 8 | AGTCATTCTGTCACGGCTTC | TCTCAACAATTGCACATTTCA |
| 9 | AAACCTACACCCCTCAAAGG | ACAAATGCACCATCCAGAGT |
| 10 | TAGCTCTCCCTGGTGTTTTG | TTTTCCACCCTCTTGTCTTT |
| 11 | TTTGTGTATGCAGTGAAGGC | TACCTACATGCGAAACATGC |
| 12 | TCAGCCCCAAATAATGTTGT | TCCATATCCAGTGAGGTGCT |

| *WNT10A* | 5’ → 3’ | |
| --- | --- | --- |
|  | F | R |
| 1 | CGCGCTCCACACACAGGCAT | GCTGCGACACACAGCTCCGA |
| 2 | GAGGCACCGGGAGTTGTCGC | GGCCGGGGTCCCAACTCTCT |
| 3 | TGGCCGTTGGGACAGAGTGTG | ATGCTGGATGGGGCAGGCAG |
| 4 | CCAGAAGCGGCTGCCC | CACAGAGGTAGGCCAGTGTC |
| 5 | GACTCTCCTGCATACTGGGC | AGAAGAGAGGTAGGCCAGGG |
