# Supplementary material for: Two novel mutations in MSX1 causing oligodontia
Source: PLoS One. 2020 Jan 8;15(1):e0227287. doi: 10.1371/journal.pone.0227287 (PMC6948825; doi:10.1371/journal.pone.0227287)
Supplement: S2 Table — (DOCX) [file pone.0227287.s004.docx]

**S2 Table. Missense /nonsense MSX1 mutations from HGMD (2019.3).**

| Mutation | | Classes of the variant | Phenotype | Excluded reasons |
| --- | --- | --- | --- | --- |
| c.65 G>A | p. G22D | DM | Cleft lip and palate | i) |
| c.95 C>T | p. A32V | DM | Oligodontia | i) |
| c.119 C>G | p. A40G | DP | Cleft lip and palate with tooth agenesis | i) |
| c.127 A>C | p. M43L | DM? | Cleft lip and palate | i) |
| c.200 T>A | p. M67K | DM | Tooth agenesis | i) |
| c.251 A>T | p. E84V | DM | Cleft lip and palate | i) |
| c.290 G>A | p. G97D | DM | Cleft lip and palate | i) |
| c.311 G>A | p. G104E | DM | Cleft lip and palate | i) |
| c.332 C>A | p. S111* | DM | Tooth agenesis and orofacial clefting | i) |
| c.348 C>T | p. G116= | DP | Oligodontia | i) |
| c.359 T>G | p. V120G | DM | Cleft lip and palate | i) |
| c.365 G>A | p. G122E | DM | Cleft lip and palate | i) |
| c.434 G>A | p. W145* | DM | Oligodontia | i) |
| c.458 C>A | p. P153Q | DM | Cleft lip and palate | i) |
| c.464 C>A | p. P155Q | DM | Hypodontia | i) |
| c.471 G>A | p. R157S | DM | Cleft lip and palate | i) |
| c.476 T>G | p. L159R | DM? | Oligodontia | i) |
| c.517 C>A | p. R173S | DM | Oligodontia | iv) |
| c.526 C>T | p. R176W | DM? | Oligodontia | iii) |
| c.539 C>T | p. T180I | DM | Hypodontia | Included |
| c.577 C>T | p. Q193* | DM | Tooth agenesis | i) |
| c.581 A>G | p. K194R | DM? | Bicuspid aortic valve | ii) |
| c.583 C>T | p. Q195* | DM | Tooth agenesis with cleft lip | i) |
| c.599 C>T | p. A200V | DM? | Oligodontia | iii) |
| c.605 G>C | p. R202P | DM | Tooth agenesis | Included |
| c.607 G>A | p. A203T | DM | Preaxial polydactyly | ii) |
| c.610 G>T | p. E204* | DM | Oligodontia | i) |
| c.623 C>A | p. S208* | DM | Witkop syndrome | i) |
| c.632 T>G | p. L211R | DM | Oligodontia | Included |
| c.668 G>T | p. R223L | DM? | Hypodontia | iii) |
| c.673 G>A | p. A225T | DM | Tooth agenesis | Included |
| c.680 C>A | p. A227E | DM | Oligodontia | Included |
| c.689 T>C | p. L230P | DM | Hypodontia | Included |
| c.739 C>T | p. P247S | DM | Preaxial polydactyly | i) |
| c.778 C>A | p. P260T | DM? | Cleft lip and/or palate | i) |
| c.818 G>C | p. G273A | DM? | Cleft lip, incomplete | i) |
| c.817 G>T | p. G273C | DM | Cleft lip with or without cleft palate, nonsydromic | i) |
| c.850 C>T | p. P284S | DM | Cleft lip with or without cleft palate, nonsydromic | i) |

Note: Based on the professional version of HGMD (2019.3), there are 38 missense/nonsense MSX1 mutations in total. Inclusion criteria: i) missense mutation in homeodomain (172nd-231st amino acids); ii) the phenotype should be hypodontia, oligodontia or tooth agenesis; iii) the classes of the variant should be “DM”; iv) Not compound with other mutations.
