# Supplementary material for: Two novel mutations in MSX1 causing oligodontia
Source: PLoS One. 2020 Jan 8;15(1):e0227287. doi: 10.1371/journal.pone.0227287 (PMC6948825; doi:10.1371/journal.pone.0227287)
Supplement: S3 Table — (DOCX) [file pone.0227287.s005.docx]

**S3 Table. The （average） number of patients and missing teeth in *MSX1* mutations with and without hydrogen**-**bonding alteration.**

| ***MSX1* mutations without** **hydrogen-bonding alteration** | | | ***MSX1* mutations with** **hydrogen-bonding alteration** | | |
| --- | --- | --- | --- | --- | --- |
| **Mutation site** | **No. of patients** | **No. of missing teeth** | **No. of missing teeth** | **No. of patients** | **Mutation site** |
| **T180I** | 1 | 5 | 10 | 1 | **F191S** |
| **L230P** | 5 | 4 | 7 | 9 | **R202P** |
|  |  | 4 | 6 |  |  |
|  |  | 4 | 4 |  |  |
|  |  | 4 | 6 |  |  |
|  |  | 2 | 6 |  |  |
|  | | | 9 |  |  |
|  |  |  | 11 |  |  |
|  |  |  | 7 |  |  |
|  |  |  | 5 |  |  |
|  |  |  | 18 | 3 | **L211P** |
|  |  |  | 8 |  |  |
|  |  |  | 9 |  |  |
|  |  |  | 11 | 2 | **A225T** |
|  |  |  | 19 |  |  |
|  |  |  | 5 | 4 | **A227E** |
|  |  |  | 9 |  |  |
|  |  |  | 5 |  |  |
|  |  |  | 13 |  |  |
| **No. of patients** 6 | | | 19 **No. of patients** | | |
| **Total missing teeth**  23 | | | 168 **Total missing teeth** | | |
| **Average number of missing teeth** 3.8 | | | 8.8 **Average number of missing teeth** | | |
