# Supplementary material for: Two novel mutations in MSX1 causing oligodontia
Source: PLoS One. 2020 Jan 8;15(1):e0227287. doi: 10.1371/journal.pone.0227287 (PMC6948825; doi:10.1371/journal.pone.0227287)
Supplement: Attachment — Submitted filename: Comments on PONE-D-19-23967.docx [file pone.0227287.s006.docx]

The manuscript by Yang et al reported two novel MSX1 mutations responsible for non-syndromic tooth agenesis. The authors also reviewed genotype-phenotype relationship of MSX1 missense mutations via 3D-structural analysis and concluded that *MSX1* mutations that altered hydrogen bonding tend to cause a more severe non-syndromic TA phenotype with more missing teeth. The analysis is based on 7 mutations identified in the published literature. Overall the study is interesting and well designed. However, there are some issues that need to be addressed before publication.

1. The author concluded that *MSX1* mutations that altered hydrogen bonding tend to cause a more severe non-syndromic TA phenotype with more missing teeth. How did they get this conclusion? Because there was not any statistical genotype-phenotype correlation analysis compared between the MSX1 mutations with or without altered hydrogen bonding. What is the average number of missing teeth in *MSX1* mutations with or without altered hydrogen bonding respectively? How many *MSX1* mutations without altered hydrogen bonding were included for the statistical analysis and the reason for selection should be well described.
2. The manuscript needs to be carefully revised and well organized. In the Discussion part, from line 211 to line 282, include the descriptions of methods, results and discussion about *MSX1* mutations that altered hydrogen bonding seems to cause more severe non-syndromic TA phenotype.
3. All the figures are of poor quality, not clear. They should provide better and higher resolution images to readers.
4. Mutational analysis was only performed in the proband of family 2. The pathogenic mutations of other members in this family are needed to be confirmed, especially his mother (II:2)，since she was also a tooth agenesis patient.
5. How did they get the results of Figure 5 should be well descripted, because that is the key result supporting their conclusion. It should be include in the main body of the manuscript.
