# Supplementary material for: Two novel mutations in MSX1 causing oligodontia
Source: PLoS One. 2020 Jan 8;15(1):e0227287. doi: 10.1371/journal.pone.0227287 (PMC6948825; doi:10.1371/journal.pone.0227287)
Supplement: Attachment — Submitted filename: Response letter.docx [file pone.0227287.s007.docx]

Dear Editor,

Thank you for your letter and the reviewers’ comments concerning our manuscript entitled “Two Novel Mutations in MSX1 Causing Oligodontia”. We have studied their comments carefully and have made correction which we hope meet their approval.

We enclosed a revised manuscript according to the comments of the reviewers. Revised portion are highlighted in yellow.

Response to editor:

1. In abstract: page 2, line 12: The phrase “with” was replaced by “between”.
2. In abstract: page 2, line 22: The phrase “ultimately” was replaced with “thereby potentially”.
3. We have rewritten the 2nd paragraph of the Introduction part as the editor’s suggestion.
4. In the Introduction part, 3rd paragraph: We have attached the references according to which we selected the five candidate genes.
5. In Results part: We have added the range of homeodomain in amino acid residue numbers (No. 172-231).
6. We didn’t make the criteria clear based on which we included the 7 MSX1 mutations in our manuscript. We will explain the criteria below and add it into the manuscript.

Based on the professional version of HGMD (2019.3)[1], there are 38 missense/nonsense MSX1 mutations in total (Table).

Inclusion criteria: i) missense mutation in homeodomain (172nd-231st amino acids) (26 mutations are excluded, red ones); We can only 3D-reconstruct the model of homeodomain, therefore, the mutation outside homeodomain was excluded.

ii) the phenotype should be hypodontia, oligodontia or tooth agenesis (2 mutations are excluded, orange ones);

iii) the classes of the variant should be “DM” (3 mutations are excluded, purple ones); “DM?”denotes a probable/ possible pathological mutation. And as to those excluded MSX1 mutation, A200V was incomplete penetrance; R223L have no detailed phenotype.

iv) Not compound with other mutations. R173S mutation was excluded, because the proband have a compound EDA mutation. (green one).

In conclusion, T180I, F191S (this study), R202P, L221R, A225T, A227E and L230P were included in our study.

Table. 38 missense /nonsense MSX1 mutations from HGMD (2019.3).

| Mutation | | Classes of the variant | Phenotype |
| --- | --- | --- | --- |
| c.65 G>A | p. G22D | DM | Cleft lip and palate |
| c.95 C>T | p. A32V | DM | Oligodontia |
| c.119 C>G | p. A40G | DP | Cleft lip and palate with tooth agenesis |
| c.127 A>C | p. M43L | DM? | Cleft lip and palate |
| c.200 T>A | p. M67K | DM | Tooth agenesis |
| c.251 A>T | p. E84V | DM | Cleft lip and palate |
| c.290 G>A | p. G97D | DM | Cleft lip and palate |
| c.311 G>A | p. G104E | DM | Cleft lip and palate |
| c.332 C>A | p. S111* | DM | Tooth agenesis and orofacial clefting |
| c.348 C>T | p. G116= | DP | Oligodontia |
| c.359 T>G | p. V120G | DM | Cleft lip and palate |
| c.365 G>A | p. G122E | DM | Cleft lip and palate |
| c.434 G>A | p. W145* | DM | Oligodontia |
| c.458 C>A | p. P153Q | DM | Cleft lip and palate |
| c.464 C>A | p. P155Q | DM | Hypodontia |
| c.471 G>A | p. R157S | DM | Cleft lip and palate |
| c.476 T>G | p. L159R | DM? | Oligodontia |
| c.517 C>A | p. R173S | DM | Oligodontia |
| c.526 C>T | p. R176W | DM? | Oligodontia |
| c.539 C>T | p. T180I | DM | Hypodontia |
| c.577 C>T | p. Q193* | DM | Tooth agenesis |
| c.581 A>G | p. K194R | DM? | Bicuspid aortic valve |
| c.583 C>T | p. Q195* | DM | Tooth agenesis with cleft lip |
| c.599 C>T | p. A200V | DM? | Oligodontia |
| c.605 G>C | p. R202P | DM | Tooth agenesis |
| c.607 G>A | p. A203T | DM | Preaxial polydactyly |
| c.610 G>T | p. E204* | DM | Oligodontia |
| c.623 C>A | p. S208* | DM | Witkop syndrome |
| c.632 T>G | p. L211R | DM | Oligodontia |
| c.668 G>T | p. R223L | DM? | Hypodontia |
| c.673 G>A | p. A225T | DM | Tooth agenesis |
| c.680 C>A | p. A227E | DM | Oligodontia |
| c.689 T>C | p. L230P | DM | Hypodontia |
| c.739 C>T | p. P247S | DM | Preaxial polydactyly |
| c.778 C>A | p. P260T | DM? | Cleft lip and/or palate |
| c.818 G>C | p. G273A | DM? | Cleft lip, incomplete |
| c.817 G>T | p. G273C | DM | Cleft lip with or without cleft palate, nonsydromic |
| c.850 C>T | p. P284S | DM | Cleft lip with or without cleft palate, nonsydromic |

7. Page 10, line 186-196, figures 3C-3D of this part was included as a supplementary figure.
8. In Discussion part, page 11, line 205-206, The phrase “demonstrate “was replaced with “show” and the phrase “leads” was replaced with “may lead”.
9. Page11, line 211-212, we have deleted the statement.
10. The same as 6^th^ answer.
11. Figure 4 was used as a supplementary figure.
12. We can only get the 3D-model of HOX domain. Therefore, MSX1 mutations in non-HOX domain were excluded in the phenotypic comparison by 3D-structure analysis.

Reference:

1.	Stenson PD, Mort M, Ball EV, Evans K, Hayden M, Heywood S, et al. The Human Gene Mutation Database: towards a comprehensive repository of inherited mutation data for medical research, genetic diagnosis and next-generation sequencing studies. Hum Genet. 2017;136(6):665-77. doi: 10.1007/s00439-017-1779-6. PubMed PMID: 28349240; PubMed Central PMCID: PMCPMC5429360.

Response to reviewers:

Reviewer #1：

1. In this study, we only included and analyzed 7 missense mutations occurred in the discussion part. And the manuscript was not construct as a review. So we think it might be better to keep the original title “Two Novel Mutations in MSX1 Causing Oligodontia”.
2. We have added the sequence diagram of the patient's mother (II2) in family 2.

Reviewer #2:

1. We reviewed 7 missense mutations in the homeodomain of *MSX1*, 2 of them did not cause hydrogen bonds alteration, and the others caused hydrogen bond alteration. We counted the number of missing teeth caused by each missense mutation, and applied Prism 5 software for statistical analysis. See the supplementary Table 3 for details. The reason why we chosen these 7 missense mutations can be seen in the 6th response to editor.
2. We have revised the parts of the manuscript you proposed to be well organized.
3. We had changed the original images to better and higher resolution ones.
4. We have added the sequence diagram of the patient's mother (II2) in family. But we can’t contact with the other family members.
5. The way how we got the result of Figure 4 （original Figure 5）was the same as to 1^st^ answer. Besides, we also added some details in the manuscript and we had already put Figure 4 （original Figure 5）in the main body of the manuscript.
